# Association Between the Meal Timing of Dietary Flavonoids and Non-Alcoholic Fatty Liver Disease in U.S. Adults: An NHANES Analysis

**DOI:** 10.3390/healthcare14121712

**Published:** 2026-06-15

**Authors:** Xiyun Ren, Shuaishuai Zhou, Yao Li

**Affiliations:** College of Animal Science and Technology, Northeast Agricultural University, Harbin 150030, China; 102306@hrbmu.edu.cn (X.R.); b230501015@neau.edu.cn (S.Z.)

**Keywords:** dietary flavonoids, non-alcoholic fatty liver disease, National Health and Nutrition Examination Survey, dietary substitution model, breakfast and dinner

## Abstract

Objective: The aim of this study was to evaluate associations between flavonoid intake at dinner versus breakfast and the odds of non-alcoholic fatty liver disease (NAFLD) using data from the National Health and Nutrition Examination Survey (NHANES). Methods: According to total flavonoids, flavonoids intake at breakfast and dinner, or flavonoids intake differences at dinner and breakfast (Δ = dinner-breakfast), we divided 3240 participants from the NHANES (2007–2010) into five groups. Logistic regression analyses were performed to examine associations between total flavonoids, flavonoid intake at breakfast and dinner or Δ flavonoids and NAFLD after the adjustment of several confounding factors. Dietary substitution models were used to estimate the association between replacing 5% of flavonoid intake at dinner with that at breakfast and the odds of NAFLD. Results: For total flavonoids intake and flavonoids intake at breakfast, when compared to the lowest quintile (Q1), participants in the highest quintile of total flavonoids intake had lower odds of NAFLD, with an adjusted odds ratio (OR) for NAFLD of 0.78 (95% confidence interval (CI): 0.61–0.98) and a *p*-trend of 0.076. Participants in the highest quintile of flavonoid intake at breakfast had an adjusted OR for NAFLD of 0.87 (95% CI: 0.69–1.09) and a *p*-trend of 0.035, also showing a trend toward lower odds of NAFLD. Conversely, for Δ flavonoids, participants in the highest quintile had higher odds of NAFLD, with an adjusted OR for NAFLD of 1.27 (95% CI: 1.01–1.60) and a *p*-trend of 0.093. When total flavonoid intake in participants remained constant, replacing 5% of dietary flavonoid intake at dinner with that at breakfast was associated with 7% lower odds of NAFLD. Conclusions: Higher flavonoid intake at breakfast than at dinner may be associated with lower odds of NAFLD. Future research should focus on the potential role of breakfast-timed flavonoid intake in preventing the onset of NAFLD.

## 1. Introduction

Non-alcoholic fatty liver disease (NAFLD) is the most prevalent liver disease caused by excessive lipid deposition in the liver; it is often accompanied by obesity, type 2 diabetes mellitus (T2DM), dyslipidemia, abnormal blood pressure, and other metabolic disorders [1]. In 2023, a multi-society Delphi consensus redefined the nomenclature from NAFLD to metabolic dysfunction-associated steatotic liver disease (MASLD) to better reflect the metabolic underpinnings of the disease. NAFLD reportedly affects approximately one in four of the global population. In the United States, NAFLD prevalence in the general North American population is estimated at approximately 24%, while it is as high as 32% in South America [2]. Thus, NAFLD has become a serious public health problem.

NAFLD pathogenesis is associated with a disrupted circadian clock combined with perturbed growth and sex hormone pathways [3]. Changes in circadian rhythms are closely related to NAFLD occurrence and development. Accumulating evidence now suggests that circadian rhythms closely influence NAFLD pathology by interfering with glucose, liver lipid and bile acid metabolism [4]. Circadian disturbances also increase circulating bile acids, especially conjugated primary bile acids, decrease secondary bile acids, and increase the ratio of cholic acid to chenodeoxycholic acid, thereby exacerbating NAFLD progression [5,6].

Dietary factors are significant adjustable factors and have critical roles in NAFLD prevention and management. Flavonoids are a ubiquitous group of naturally occurring polyphenolic compounds with remarkable roles in preventing some chronic diseases. Flavonoids prevented or even treated circadian-related diseases and functioned as circadian modulators [7]. Flavonoids also enhanced circadian rhythm amplitude levels in diet-induced obese mice [8], as amplitude reductions are generally harmful to living organisms [7].AMP-activated protein kinase (AMPK), widely recognized as the “metabolic master switch” of hepatic lipid metabolism, coordinates fatty acid oxidation and lipogenesis by phosphorylating ACC, suppressing SREBP-1c, and activating PPARα/CPT-1 [9,10,11]. In NAFLD, hepatic AMPK activity is typically markedly attenuated, directly shifting lipid metabolism from catabolism toward anabolism and serving as a key driver of excessive lipid accumulation. Notably, AMPK also serves as a molecular bridge linking metabolic signaling to the circadian clock: AMPK directly phosphorylates CRY1, triggering its degradation and thereby resetting the CLOCK: BMAL1 transcriptional loop [12]. Consistent with this, AMPK activity exhibits robust circadian oscillation, peaking during the daytime/active phase [13]. Flavonoids exert their anti-NAFLD effects primarily through AMPK activation [14], and a landmark 2025 study in Cell Metabolism demonstrated that AMPK activator C29 administered at ZT12 (biological dawn) significantly outperformed ZT0 dosing in enhancing exercise capacity and metabolic outcomes—providing direct evidence that flavonoid intake during the daytime AMPK-activity window maximizes the protective effect against NAFLD [13].

Currently, the effects of dietary factors (including nutrient and intake time) and circadian rhythmicity are clear in this new research area called “chrononutrition” [15]. However, the relationships between the specific time at which flavonoids are ingested (for instance, whether they are consumed in the morning or evening) and the odds of NAFLD remain largely unclear. This lack of clarity is mainly due to the limited research focus on the temporal aspect of flavonoid intake in the existing literature. Therefore, there is an urgent and pressing need for in-depth research to comprehensively elucidate the potential impact of flavonoid intake time on the odds of NAFLD, which could provide valuable insights for the development of more targeted dietary intervention strategies.

To address this, we used NHANES (2007–2010) data to examine the relationship between flavonoid intake at dinner versus breakfast and the odds of NAFLD, providing a theoretical basis for NAFLD prevention.

## 2. Methods

### 2.1. Study Population

NHANES is a population-based survey designed to collect information on the health and nutritional status of household populations in the U.S., and was approved by the National Center for Health Statistics Research Ethics Review Board. Detailed NHANES information was previously provided [16]. All databases can be obtained from the NHANES website (https://wwwn.cdc.gov/nchs/nhanes/Default.aspx, (accessed on 7 September 2025), and all participants provided informed consent.

In total, 18,207 participants were included in the NHANES database (2007–2010). Participants were excluded when flavonoid data were missing from breakfast or/and dinner (*n* = 6691). Also excluded were individuals < 18 years old (*n* = 4093), pregnant women (*n* = 77), and individuals with total energy intake <500 kcal/day or >4500 kcal/day (*n* = 102). Participants were also excluded when NAFLD diagnostic data were missing (*n* = 4004). Finally, the data from 3240 participants were analyzed.

### 2.2. Dietary Assessment

A 24 h dietary recall survey was used to obtain food intake on 2 non-consecutive days. The first 24 h dietary recall was conducted in person, and the second 24 h dietary recall was conducted by telephone 3 to 10 days later. Participants were asked to provide the consumption time for each food and drink during the interviews. Dietary flavonoid intake was estimated using the USDA Dietary Study Food and Nutrient Database. For each participant, the amount of each food intake was listed according to the mealtimes.

### 2.3. Main Exposures

The exposure variables included total flavonoids intake, flavonoids intake at breakfast or dinner, and differences in flavonoids intake between dinner and breakfast (Δ = dinner-breakfast).

### 2.4. Study Outcomes

The outcome of interest was NAFLD. It was defined using the United States fatty liver index (USFLI), which was established in the NHANES database and validated in multiple previous epidemiological studies [17]. Although the USFLI is a prediction model rather than a diagnostic gold standard, previous studies have demonstrated that the USFLI predicted NAFLD with an AUC (95% CI) of 0.80 (0.77–0.83). At 90% specificity, the USFLI achieved a sensitivity of 0.56, and at 90% sensitivity, it achieved a specificity of 0.34, indicating its moderate diagnostic performance as a screening tool for NAFLD. USFLI was calculated using the following equation: USFLI = (e^−0.8073×non-Hispanic blacks+0.3458×Mexican Americans+0.0093×age+0.6151×ln(gamma-glutamyltransferase)+0.0249×waist circumference+1.1792×ln(insulin)+0.8242×ln(glucose)−14.7812^)/(1 + e^−0.8073×non-Hispanic blacks+0.3458×Mexican Americans+0.0093×age+0.6151×ln(gamma-glutamyltransferase)+0.0249×waist circumference+1.1792×ln(insulin)+0.8242×ln(glucose)−14.7812^) × 100. A USFLI ≥ 30 score indicated NAFLD [17].

### 2.5. Covariate Assessments

Confounding variables included sex (male, female), age (years), race (Mexican American, other Hispanic, non-Hispanic White, non-Hispanic Black, and others), smoking status (currently smoking, non-smoking, given up smoking), drinking status (drinks/week), education level (<9th grade, 9–11th grade, high school graduate/general educational development or equivalent, college or Associate in Arts degree, college graduate or above), annual household income (≥$20,000, <$20,000), marital status (married/living with partner, widowed/divorced/separated, or never married), total energy (kcal/day), protein (g/day), fat (g/day), carbohydrates (g/day), monounsaturated fatty acid (MUFA, g/day), polyunsaturated fatty acid (PUFA, g/day), saturated fatty acid (SFA, g/day), body mass index (BMI, kg/m^2^). BMI was calculated as weight (kg) divided by height (m) squared. The alternative healthy eating index (AHEI) was developed from the original Healthy Eating Index, which included 11 food components identified through a comprehensive review of studies.

### 2.6. Statistical Analysis

All statistical analyses were performed using R 3.5.3 (www.r-project.org/, accessed on 5 January 2025). A two-sided *p* < 0.05 value was considered statistically significant. Continuous variables were described by the mean ± standard deviation, and categorical variables by percentages. Missing covariables (<5%) were filled in by multiple interpolation.

After total flavonoids intake and flavonoids intake values at breakfast or dinner were categorized into quintiles, multivariable logistic regression models were performed to explore independent associations with the odds of NAFLD, with the lowest quintile considered the reference. Adjusted covariates in models included age, sex, race, smoking status, drinking status, education level, annual household income, marital status, total energy, protein, fat, carbohydrates, MUFA, PUFA, and AHEI. Additionally, according to differences (Δ) in flavonoid intake between dinner and breakfast, Δ flavonoids were categorized into quintiles, and multivariable logistic regression models were then performed to explore the independent association of Δ with NAFLD after adjustment for several confounding factors.

In nutritional epidemiology, food substitution models have been used to study the relationship between nutrient or food substitution and related health or disease outcomes and to offer dietary advice for the prevention and treatment of diseases. The dietary substitution model is a new statistical method that holds total flavonoids intake constant to evaluate changes in the odds of NAFLD with a theoretical shift of the flavonoids from one time period to another [18]. In this study, the method of substitution analysis is used to study the substitution of flavonoids at dinner with flavonoids at breakfast under the premise of equal flavonoid intake, and to observe changes in epidemiological indicators. We estimated the associations between replacing 5% of flavonoid intake at dinner with flavonoid intake at breakfast and the odds of NAFLD.

### 2.7. Sensitivity Analysis

To verify the stability of our research results, two sensitivity analyses were conducted. First, total flavonoids, flavonoids at breakfast or dinner and flavonoid differences at dinner versus breakfast were adjusted by energy, and the data were reanalyzed. Second, total flavonoids, flavonoids at breakfast or dinner and flavonoid differences at dinner versus breakfast were adjusted by BMI, and the data were reanalyzed.

### 2.8. Subgroup Analysis

This study further investigated the associations of flavan-3-ols (mg) or flavonols (mg) consumed at breakfast or dinner, total flavan-3-ols or flavonols, as well as the difference between dinner and breakfast intake, with the odds of NAFLD after controlling for several confounding factors, including age, sex, race, smoking status, drinking status, education level, annual household income, marital status, total energy, protein, fat, carbohydrates, MUFA, PUFA and AHEI.

## 3. Results

### 3.1. Baseline Characteristics

NHANES provided data on 3420 individuals. Both demographics and nutrition characteristics with respect to total flavonoid levels in quintiles were shown (Table 1). Age, race, smoking status, drinking status, education level, marital status, total energy, protein, fat, carbohydrates, MUFA, PUFA, AHEI, total flavonoids intake, flavonoids intake at breakfast or dinner, and Δ flavonoids were significantly different across quintiles (1–5) (*p* < 0.05). However, sex, marital status, annual household income, and total SFA intake were not significantly different across quintiles (*p* > 0.05).

### 3.2. Associations Between Flavonoid Intake (Total, at Breakfast, and at Dinner) and the Odds of NAFLD

**Figure 1:**
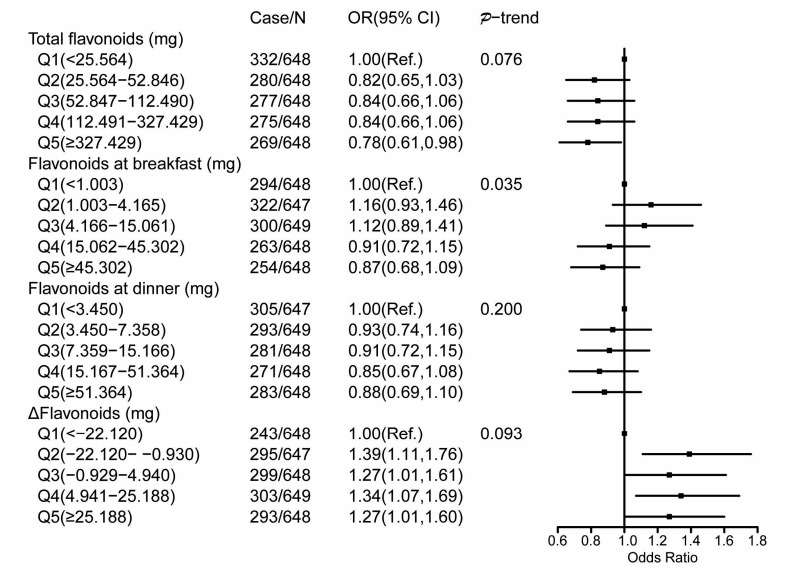
Associations of total flavonoids intake, flavonoids intake at breakfast or dinner, and Δ flavonoids intake with the odds of NAFLD by logistic regression models (*n* = 3240). Models were adjusted for age, sex, race, smoking status, drinking status, education level, annual household income, marital status, total energy, protein, fat, carbohydrates, MUFA, PUFA, and AHEI.

These associations were examined in multivariable logistic regression models (Figure 1) and suggested a negative relationship between total flavonoids intake and flavonoids intake at breakfast and the odds of NAFLD after controlling for several confounding factors, including age, sex, race, smoking status, drinking status, education level, annual household income, marital status, total energy, protein, fat, carbohydrates, MUFA, PUFA and AHEI. For total flavonoids intake and flavonoids intake at breakfast, compared to the lowest quintile (Q1), adjusted NAFLD odds ratios (ORs) in the highest quintile (Q5) were 0.78 (95% confidence interval (CI): 0.61–0.98) and 0.87 (95% CI: 0.68–1.09), and *p* trends were 0.076 and 0.035, respectively. Segmented regression analysis revealed a threshold of 114.847 mg for flavonoid intake at breakfast, below which the odds of NAFLD were higher. However, for flavonoid intake at dinner, the adjusted NAFLD OR in Q5 was 0.88 (95% CI: 0.69–1.10), and the *p* trend was 0.200. These results indicated that flavonoid intake at dinner was not significantly associated with the odds of NAFLD.

### 3.3. Associations Between Flavonoid Intake at Dinner Versus Breakfast and the Odds of NAFLD

The associations of Δ flavonoids with NAFLD were examined using multivariable logistic regression models (Figure 1). High Δ flavonoids were associated with higher odds of NAFLD after adjusting for confounding factors. When compared to Q1, the adjusted NAFLD OR in Q5 was 1.27 (95% CI: 1.01–1.60), and the *p* trend was 0.093.

### 3.4. Dietary Substitution Models

**Figure 2:**
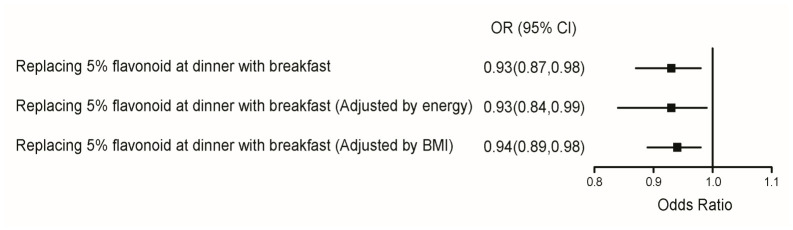
Adjusted ORs for NAFLD: substitution of 5% flavonoids intake from dinner to breakfast. Adjusted by age, gender, education, smoking, drinking, marital status, household income, total energy, protein, fat, carbohydrate, SFA, MUFA, PUFA and AHEI. ORs represent the odds of NAFLD 5% substitution of flavonoid intake from dinner to breakfast (reference: no substitution). OR < 1 indicates reduced NAFLD risk when flavonoids are consumed at breakfast rather than dinner.

The dietary substitution model results were presented in Figure 2. Substituting 5% of flavonoid intake at dinner with that at breakfast was associated with 7% lower odds of NAFLD (OR = 0.93, 95% CI: 0.87–0.98). Additionally, after adjusting for flavonoid intake at breakfast and dinner for energy or BMI, the odds of NAFLD were 7% and 6% lower, respectively (OR = 0.93, 95% CI: 0.84–0.99 and OR = 0.94, 95% CI: 0.89–0.98).

### 3.5. Sensitivity Analysis

**Figure 3:**
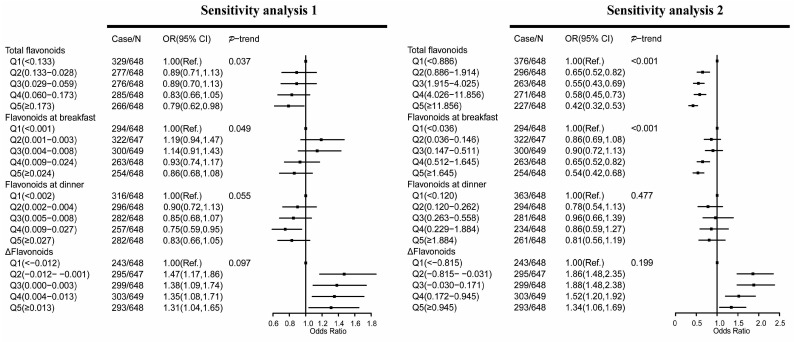
Sensitivity analysis 1: Associations of total flavonoids intake, flavonoids intake at breakfast or dinner, and Δ flavonoids intake adjusted for total energy with the odds of NAFLD by logistic regression models (*n* = 3240). Sensitivity analysis 2: Associations of total flavonoids intake, flavonoids intake at breakfast or dinner, and Δ flavonoids intake adjusted for BMI with the odds of NAFLD by logistic regression models (*n* = 3240). Models were adjusted for age, sex, race, smoking status, drinking status, education level, annual household income, marital status, total energy, protein, fat, carbohydrates, MUFA, PUFA, and AHEI.

Detailed sensitivity analyses are shown (Figure 3). After total flavonoids intake and flavonoids intake at breakfast were adjusted by energy, and when compared to Q1, adjusted NAFLD ORs in Q5 were 0.79 (95% CI: 0.62–0.98) and 0.86 (95% CI: 0.68–1.08), and *p* trends were 0.037 and 0.049, respectively. Through segmented regression, a threshold effect was observed for total energy-adjusted flavonoid intake at breakfast (0.062), below which the odds of NAFLD were higher. After total flavonoids intake and flavonoids intake at breakfast were adjusted by BMI, adjusted NAFLD ORs in Q5 were 0.42 (95% CI: 0.32–0.53) and 0.54 (95% CI: 0.42–0.68), and *p* trends were <0.001 and <0.001, respectively. Through segmented regression, a threshold effect was observed for total BMI-adjusted flavonoids intake at breakfast (0.916), below which the odds of NAFLD were higher. When Δ flavonoids were adjusted by energy or BMI and compared to Q1, adjusted NAFLD ORs in Q5 were 1.31 (95% CI: 1.04–1.65) and 1.34 (95% CI: 1.06–1.69), and *p* trends were 0.097 and 0.199, respectively. Similarly, in both sensitivity analyses, for flavonoid intake at dinner, adjusted NAFLD ORs in Q5 were 0.83 (95% CI: 0.66–1.05) and 0.81 (95% CI: 0.56–1.19), and *p* trends were 0.055 and 0.477, respectively.

### 3.6. Subgroup Analysis

**Figure 4:**
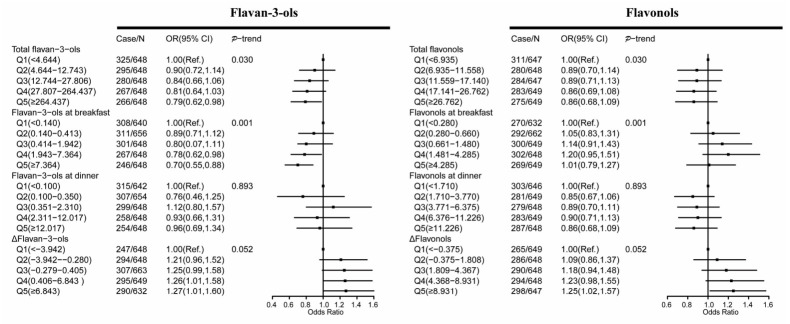
Associations of total flavan-3-ols or flavonols intake, flavan-3-ols or flavonols intake at breakfast or dinner, and Δ flavan-3-ols or Δ flavonols intake adjusted for total energy or BMI with the odds of NAFLD by logistic regression models (*n* = 3240). Models were adjusted for age, sex, race, smoking status, drinking status, education level, annual household income, marital status, total energy, protein, fat, carbohydrates, MUFA, PUFA, and AHEI.

The detailed results of the subgroup analyses were shown in Figure 4. Multivariable logistic regression models demonstrated that Δ flavan-3-ols or Δ flavonols were positively associated with NAFLD. Compared to the lowest quintile, adjusted NAFLD odds ratios in the highest quintile were 1.27 (95% CI: 1.01–1.60) and 1.25 (95% CI: 1.02–1.57), and *p* trends were 0.052 and 0.300, respectively. The results of subgroup analyses were consistent with the primary findings of this study.

## 4. Discussion

To the best of our knowledge, ours is the first study to examine associations between flavonoid intake at breakfast and dinner and NAFLD incidence using a nationally representative sample from the U.S. These findings indicated that greater flavonoid intake at dinner compared with breakfast was associated with higher odds of NAFLD and highlighted the importance of a proper flavonoid distribution at breakfast and dinner throughout the day. In 2023, a multi-society Delphi consensus redefined the nomenclature from NAFLD to MASLD to better reflect the metabolic underpinnings of the disease. Throughout this study, we retain the term NAFLD for consistency with the original diagnostic criteria applied at the time of data collection.

Previous studies reported that flavonoids improved cholesterol metabolism and insulin resistance, alleviated oxidative damage, and also reduced hepatic triglyceride accumulation, which potentially prevented NAFLD occurrence [19,20]. A reverse significant association was also identified between flavonoid consumption and the odds of NAFLD [21]. Flavonoids also exert hepatoprotective effects and may become effective drugs for NAFLD treatment in the future [22]. Using a large nationally representative sample, we found that higher total flavonoids intake was associated with lower odds of NAFLD.

Compared with breakfast, higher dietary flavonoid intake at dinner was associated with higher odds of NAFLD. Diets can affect circadian rhythms, and meal timing is a major factor in mammalian peripheral clocks [23]. Circadian rhythms are closely related to NAFLD, and a lack of coordination in central and peripheral circadian rhythms is a core feature in metabolic syndrome, including almost all genetic, dietary, or environmentally related NAFLD models [24]. Lipid metabolism processes closely related to NAFLD occurrence are regulated by the body’s biological clock and indicate that circadian rhythms participate in NAFLD occurrence and development by regulating lipid metabolism. Recent studies reported that flavonoids altered the body’s circadian rhythms, and their impact on circadian rhythms had common characteristics but varying abilities [7], possibly due to different action mechanisms. AMPK activity peaks during the active/morning phase, driving fatty acid oxidation and suppressing SREBP-1c-mediated lipogenesis [25,26], whereas during the rest/night phase, AMPK activity troughs, lipogenic genes (FASN, ACC, DGAT) are fully activated, and the body shifts to an energy-storage mode [27].AMPK directly phosphorylates CRY1, triggering its degradation and thereby resetting the CLOCK:BMAL1 transcriptional loop. Flavonoids exert their anti-NAFLD effects primarily through AMPK activation. The core circadian heterodimer BMAL1:CLOCK binds E-box elements to drive rhythmic Rev-erbα expression, which recruits HDAC3 to suppress Cyp7a1—the rate-limiting enzyme of bile acid synthesis—thereby gating bile acid production to the active phase [28,29]. Conversely, RORα activates Pparα during the active phase, inducing Cpt1a-mediated fatty acid β-oxidation [30]. Hepatocyte-specific Bmal1 ablation abolishes these rhythms, resulting in impaired fatty acid oxidation, hepatic triglyceride accumulation, and fasting hypoglycemia [12].

Flavonoids (e.g., rutin, hesperetin, myricetin, and naringin) may affect circadian rhythm amplitude levels. Animal studies indicated that rutin enhanced this amplitude in diet-induced obese mice; rutin acted as a receptor-related orphan receptor agonist, enhancing amplitude viaPER2 expression [8].In a fruit fly model, naringin enhanced circadian rhythm amplitude, and hesperetin prolonged the circadian rhythm cycle via its antioxidant effects. Thus, flavonoids have important roles in NAFLD occurrence and development by altering circadian rhythms. In the current study, higher flavonoid intake at dinner versus breakfast was associated with higher odds of NAFLD, which meant that different flavonoid intake timings may affect disease occurrence by altering circadian rhythms. Moreover, replacing 5% of flavonoids intake at dinner with that at breakfast was associated with 7% lower odds of NAFLD, suggesting that the timing of flavonoids intake may be relevant to the odds of NAFLD.

Disrupted circadian rhythms may also contribute to inflammation, which is associated with higher odds of NAFLD [31,32]. Macrophages are key mediators between disrupted circadian rhythms and systemic inflammation. The circadian clock REV-ERBα is a core circadian clock component, and it mediates inflammatory cytokine regulation and is proposed as a dominant regulator in hepatic lipid metabolism [33]. REV-ERBα suppresses C-C-motif chemokine ligand 2-activated intracellular mitogen-activated protein kinase pathways, including extracellular signal-regulated kinase and p38, resulting in impaired cell adhesion and macrophage migration, thus aggravating inflammation [34]. Inflammatory mechanisms are also involved across the entire NAFLD spectrum. Inflammation serves as the central engine driving NAFLD progression. During the NAFL stage, lipotoxicity activates the NLRP3 inflammasome, leading to the release of IL-1β and IL-18 and igniting the initial inflammatory response [35]. In the NASH stage, necroinflammation recruits macrophages, resulting in sustained release of TNF-α and IL-6, which activate hepatic stellate cells and promote extensive collagen deposition [36]. During the progression from fibrosis to cirrhosis, chronic inflammation drives continuous extracellular matrix accumulation and disruption of hepatic lobular architecture, ultimately increasing the risk of hepatocellular carcinoma [37]. Inflammation persists throughout all stages and represents the critical irreversible turning point in disease progression. Similarly, inflammation triggers are rooted in hepatic (lipid overload, lipotoxicity, and oxidative stress) and extrahepatic systems (gut-liver axis, adipose tissue, and skeletal muscle), resulting in unique immune-mediated NAFLD pathomechanisms [33].

Mechanistically, gut microbiota alterations may also explain our observations. Growing evidence now suggests that a disrupted gut microbiota may contribute to liver disease, including NAFLD [38]. Gut microbiota changes and elevated intestinal permeability increase liver exposure to gut-derived bacterial products, leading to chronic endotoxemia and associated gut-liver axis changes that increase NAFLD risk [39,40]. Previous research has suggested that natural compounds such as dietary flavonoids may help regulate gut microbiome composition, suggesting prebiotic capabilities and therapeutic potential for NAFLD [41]. Quercetin exerted positive effects on intestinal flora composition at different classification levels; it reduced elevated *Firmicutes*/*Bacteroidetes* ratios in NAFLD, increased Gram-negative *Proteobacteria* levels, and increased total bacterial concentrations. At class levels, NAFLD-related *Clostridia*, *Bacilli*, and *Deltraproteobacteria* were increased, and *Bacteroidia*, *Erysipelotrichi*, and *Betaproteobacteria* levels reverted to control values when rats were supplemented with quercetin to offset an imbalanced intestinal flora and potentially improve NAFLD [42]. The gut microbiota may also exhibit diurnal variations in terms of relative abundance and function to serve as host circadian rhythm and metabolism drivers. The diurnal oscillations of the gut microbiota are profoundly disrupted in NAFLD [43]. During the active/day phase, *Lactobacillus* dominates and short-chain fatty acids (SCFAs) peak (12:00 pm–03:00 am), reinforcing intestinal barrier integrity and suppressing hepatic lipogenesis [44,45]. Conversely, at night, Firmicutes and Bacteroidetes surge, driving energy harvest and de novo lipid synthesis [45]. Critically, ~61% of NAFLD patients harbor high-alcohol-producing *Klebsiella pneumonia* (HiAlcKpn), whose ethanol-producing activity and abundance escalate during the rest phase, disrupting the gut–liver axis [46]. Circadian disruption further collapses these rhythms, depleting anti-inflammatory taxa (*Ruminococcaceae*, *Akkermansiaceae*) while enriching pro-inflammatory *Proteobacteria* and *Enterobacteriaceae*, accelerating NAFLD progression [43]. Suppressed hepatic bile acid signaling further exacerbates metabolic dysfunction in NAFLD [47], whereas time-restricted feeding can ameliorate NAFLD through gut microbiota remodeling [48]. Changes in microbial oscillators under different diets may also affect host metabolism by altering central and peripheral host circadian clock functions and/or by directly affecting metabolism.

Our study was significant and innovative for the following reasons. First, we emphasized the importance of flavonoid intake times, which are not only related to considering intake at meals but also to meal timings. Moreover, we adopted novel statistical and dietary substitution models to explore changes in the odds of NAFLD when a certain proportion of flavonoids consumed at dinner were replaced by those consumed at breakfast, aiming to provide a dietary basis for effectively reducing NAFLD incidence. However, several study limitations were identified. First, the nature of this study was cross-sectional, so causal associations between chrononutrition and NAFLD were not established. Second, some confounding factors, which were not measured or recognized, may have influenced our results. Third, since NAFLD was not diagnosed using a gold standard (e.g., liver biopsy or MRI-PDFF) but rather defined by the USFLI in the present study, and given that the NHANES population comprises five distinct racial/ethnic groups, the USFLI may overestimate or underestimate NAFLD prevalence in certain ethnic subgroups, thereby introducing potential outcome misclassification bias that could affect the validity of our findings. Fourth, since flavonoids are predominantly found in plant-based foods, their intake is substantially influenced by seasonal and other environmental factors. Moreover, the NHANES dietary survey captures only two non-consecutive days of food intake, which may not accurately reflect habitual consumption patterns. These limitations could potentially introduce bias into the study findings. Fifth, the present study focused on flavonoid intake at breakfast and dinner, whereas flavonoids consumed during snacks and lunch may influence the results of the present study. Finally, owing to the lack of circadian rhythm-related variables in the database, we were unable to explore the mediating or moderating effects of circadian rhythms on the association between flavonoid intake at different time points and the odds of NAFLD.

## 5. Conclusions

Higher flavonoid intake at breakfast than at dinner may be associated with lower odds of NAFLD. Future research should focus on the potential role of breakfast-timed flavonoid intake in preventing the onset of NAFLD.

## Figures and Tables

**Table 1:** Baseline characteristics in terms of quintiles of differences in total flavonoids: NHANES, 2007–2010.

	Q1	Q2	Q3	Q4	Q5	*p*-Value
Case/*n*	332/648	280/648	277/648	275/648	269/648	
Age (years)	51.47 (17.43)	50.90 (17.64)	52.75 (18.00)	52.5 (17.26)	54.03 (16.15)	0.011
Gender (Men)	300 (48.46)	288 (44.44)	305 (47.07)	309 (47.69)	300 (46.30)	0.945
Current smoking [*n*, (%)]	95 (25.93)	113 (17.44)	91 (14.04)	95 (14.66)	95 (14.66)	<0.001
Drinking (drinks/week)	4.38 (39.21)	2.74 (2.34)	5.94 (55.41)	2.54 (2.31)	2.41 (2.09)	<0.001
College graduate or above [(*n*, (%)]	207 (15.59)	133 (20.52)	175 (27.01)	192 (29.63)	207 (31.94)	<0.001
Married [(*n*, (%)]	400 (56.17)	360 (55.56)	352 (54.32)	385 (59.41)	400 (61.73)	0.691
>$20,000 annual household income [(*n*, (%)]	518 (74.69)	498 (76.85)	493 (76.08)	497 (76.70)	518 (79.94)	0.137
Total energy (kcal/day)	1827.33 (696.40)	1992.85 (728.90)	2087.67 (730.04)	2068.19 (768.97)	2097.67 (737.50)	<0.001
Total protein (g/day)	74.38 (32.25)	78.99 (30.73)	83.58 (33.33)	82.27 (32.41)	82.02 (31.99)	<0.001
Total fat (g/day)	72.68 (34.58)	74.47 (34.30)	74.48 (33.79)	74.94 (34.81)	78.84 (35.55)	0.006
Total carbohydrate (g/day)	215.45 (90.53)	244.43 (93.82)	262.94 (91.12)	259.89 (102.72)	260.17 (97.64)	<0.001
Total SFA (g/day)	23.73 (12.35)	24.45 (12.45)	24.22 (12.31)	23.85 (12.36)	25.02 (12.21)	0.241
Total MUFA (g/day)	26.84 (13.54)	27.12 (13.23)	27.08 (12.95)	27.31 (13.46)	29.01 (14.22)	0.013
Total PUFA (g/day)	15.66 (8.31)	16.31 (8.55)	16.41 (8.35)	17.07 (9.20)	17.85 (9.28)	<0.001
Total flavonoids intake (mg/day)	14.49 (6.32)	38.10 (7.55)	78.68 (16.83)	199.96 (62.40)	747.13 (547.85)	<0.001
Flavonoids at breakfast (mg/day)	3.01 (3.95)	9.39 (9.84)	23.49 (21.48)	46.48 (62.35)	153.55 (221.16)	<0.001
Flavonoids at dinner (mg/day)	5.64 (4.76)	10.74 (9.07)	21.13 (21.50)	57.44 (72.84)	186.65 (246.54)	<0.001
Δ Flavonoids (mg/day	2.64 (6.59)	1.35 (1.49)	2.35 (3.51)	10.95 (1.07)	33.10 (3.46)	<0.001
BMI (kg/m^2^)	29.43 (6.58)	28.51 (6.11)	28.73 (5.71)	28.34 (6.48)	28.61 (6.04)	<0.001
AHEI	28.14 (7.64)	29.30 (7.22)	30.46 (7.52)	31.65 (7.79)	30.95 (8.53)	<0.001

Continuous variables are expressed as mean (SD); Categorical variables are expressed as *n* (%); Generalized linear models and χ^2^ test were used to probe for differences in continuous variables and categorical variables; Q, quintile. MUFA, monounsaturated fatty acid; PUFA, polyunsaturated fatty acid; SFA, saturated fatty acid; BMI, body mass index; T2DM, Type 2 diabetes mellitus; CVD, cardiovascular disease; AHEI, alternative healthy eating index.

## Data Availability

Publicly available datasets were analyzed in this study. This data can be found here: https://wwwn.cdc.gov/nchs/nhanes/Default.aspx, accessed on 7 September 2025.
